# Comprehensive profiling and molecular characterization of alternative splicing regulation in synaptic remodelling associated with neuropathic pain induced by chronic constrictive injury in a rat model

**DOI:** 10.1080/15476286.2026.2675080

**Published:** 2026-05-15

**Authors:** Jun Zhou, Yifan Jia, Wei Song, Ying Zhang, Zhongyuan Xia

**Affiliations:** a Department of Pain, Renmin Hospital of Wuhan University, Wuhan, Hubei Province, China; b Department of Pharmacy, Renmin Hospital of Wuhan University, Wuhan, Hubei Province, China; c Department of Vascular Surgery, Renmin Hospital of Wuhan University, Wuhan, Hubei Province, China; d Department of Anesthesiology, Renmin Hospital of Wuhan University, Wuhan, Hubei Province, China

**Keywords:** Alternative splicing, synaptic remodeling, neuropathic pain, transcriptomic profiling, RNA sequencing, RNA-binding proteins

## Abstract

Neuropathic pain (NP) is a chronic pain condition caused by injury to the nervous system. Although alternative splicing dysregulation has been implicated in NP, the contribution of RNA-binding proteins (RBPs) to this process remains unclear. We analysed 12 RNA-seq datasets (GSE217932) using the SUVA pipeline to identify splicing alterations in Sham, chronic constriction injury (CCI) and CCI treated with L-tetrahydropalmatine (CCI_THP) groups. Co-expression and co-disturbance analyses were performed to define splicing-associated RBP networks. Key findings were further validated in a CCI rat model by behavioural testing, junction-specific RT-qPCR, immunohistochemistry and immunofluorescence. Distinct splicing profiles were observed among the three groups. Differentially spliced genes were enriched in pathways related to axonogenesis, cell junction assembly and synapse assembly. Several genes, including *Lrrc4c, Shank2, Nefh, and Grid2*, showed aberrant splicing in CCI, which was partially reversed by THP treatment. K-means clustering supported a role for THP in restoring synaptic remodelling-related splicing alterations. In addition, *Nefh* splicing was associated with *Rbm47 and Grn* expression, and multiple RBPs, including *Rbm47, Grn, Lcp2, Plek, Ptpn6 and Hcls1*, were significantly upregulated in CCI. These results were supported by in vivo validation of selected splicing events and protein changes. This study provides a transcriptome-wide view of alternative splicing and RBP dysregulation in NP, and suggests that THP may alleviate neuropathic pain partly by modulating aberrant splicing programs. These findings offer insight into NP mechanisms and identify potential therapeutic targets.

## Introduction

Neuropathic pain (NP) is defined by pain originating from primary damage or dysfunction within the nervous system. It is commonly associated with lesions or diseases impacting the somatosensory nervous system, constituting a prevalent form of chronic pain that significantly impairs quality of life. Notable examples include trigeminal neuralgia, painful polyneuropathy, postherpetic neuralgia and central post-stroke pain. Patients often report persistent or intermittent spontaneous pain, characterized by sensations of burning, tingling and squeezing, which may be intensified by stimuli such as light touch and cold. This type of pain is frequently associated with conditions like nerve terminal neuromas, compressed nerves or nerve roots, dorsal root ganglia and ectopic thalamic activity, all of which may contribute to spontaneous pain in various contexts. Furthermore, evocative pain can propagate to adjacent regions through pathophysiological mechanisms involving peripheral and central sensitization. Current first-line clinical treatments for neuropathic pain primarily target the nervous system and demonstrate limited therapeutic efficacy. Emerging research suggests that neuropathic pain results from an interplay between the sensory nervous system and the associated immune system [1,2]. Current first-line clinical interventions for neuropathic pain predominantly target the nervous system and exhibit limited therapeutic efficacy. Emerging research indicates that neuropathic pain arises from a complex interaction between the sensory nervous system and the immune system.

The somatosensory injury perception system comprises complex networks that include peripheral sensory neurons, the dorsal horn of the spinal cord and the brainstem, as well as various brain regions that collectively facilitate the intricate, multidimensional experience of pain. Within this pathway, excitatory transmission at critical synapses is fundamental for the differentiation of pain. A defining feature of this system is its plasticity, which refers to its ability to adapt in response to experiential influences. Acute physiological pain serves as an alarm mechanism within the body, characterized by a high activation threshold and generally transient duration. Nevertheless, factors like tissue or nerve damage, inflammation, metabolic disorders and cancer can cause plastic changes in nociceptive pathways, turning physiological pain into chronic pathological pain. This shift involves increased pain sensitivity, lowered pain thresholds and persistent pain. Synaptic plasticity is central to these changes, affecting the balance of excitation and inhibition in nociceptive pathways [1,3].

Alternative RNA splicing represents a fundamental mechanism that augments the coding capacity of the genome. A single RNA-binding protein has the capability to regulate alternative splicing decisions across a multitude of RNA transcripts, thereby affecting the abundance and functionality of various cellular proteins. In the context of the nervous system, alternative splicing is essential for modulating neuronal function and diversity. Alternative splicing generates diverse proteins involved in various neuronal processes like synapse formation and signalling. Notably, the SLM2 splicing program specifically targets certain mRNAs for synaptic proteins. Correcting just one SLM2-dependent exon in the synapse molecule neurexin-1 can restore synaptic plasticity and reduce behavioural issues in SLM2 knockout mice [4]. To date, alternative splicing has been recognized as a critical regulatory mechanism in the pathogenesis of various diseases, including neuropathic pain. For example, research has identified two isoforms of VEGF-A in the spinal cord: the pro-pain VEGF-Axxxa and the anti-pain VEGF-Axxxb. These isoforms are regulated by the splicing factor SRSF1, suggesting that the regulation of VEGF-A through alternative splicing may contribute to neuropathic pain [5]. In conclusion, the diverse isoforms produced through alternative splicing regulation play a significant role in the context of neuropathic pain. Nevertheless, the current research on the regulatory mechanisms governing alternative splicing in neuropathic pain remains inadequate and necessitates comprehensive and extensive investigation.

## Material and methods

### Retrieval and process of public data

Public sequence data files of 12 RNA-seq data from GSE217932 were downloaded from the Sequence Read Archive (SRA). SRA Run files were converted to fastq format with NCBI SRA Tool fastq-dump. The raw reads were trimmed of low-quality bases using a FASTX-Toolkit (v.0.0.13; http://hannonlab.cshl.edu/fastx_toolkit/). Then the clean reads were FastQC (http://www.bioinformatics.babraham.ac.uk/projects/fastqc).

### Alternative splicing analysis using SUVA

The alternative splicing events and regulated alternative splicing events (RAS) among Sham, CCI and CCI_THP (L-Tetrahydropalmatine) samples were defined and quantified using the SUVA pipeline as described previously [6]. Splicing ratio difference and Reads proportion of SUVA AS event (pSAR) of each AS events were calculated.

### Identification of differentially expressed RBPs

The software DEseq2 [7], which is specifically used to analyse the differential expression of genes, was applied to screen the raw count data for DEGs. The results were analysed based on the fold change (FC ≥ 2 or ≤0.5) and false discovery rate (FDR ≤ 0.05) to determine whether a gene was differentially expressed. Then expression profile of differentially expressed RBPs were filtered out from all DEGs according to A catalogue of 1914 RNA-binding proteins (RBPs) of mouse was retrieved from previous report [8].

### Functional enrichment analysis

To identify functional categories of genes, we employed the clusterProfiler package (v4.6.2) [9], which enabled us to determine Gene Ontology (GO) terms and KEGG pathways.

### Co-expression analysis

The co-expression between splicing ratio of RAS and expression of its host gene was established. |Pearson’s correlation coefficient| ≥0.8 and *p* value ≤0.01 were demonstrated. The co-disturbed network between the expression of DE RBPs and the splicing ratio of RAS was constructed, with a |Pearson’s correlation coefficient| ≥0.85 and *p* value ≤0.01 being retained.

### Statistical analysis

Principal component analysis (PCA) analysis was performed by R package factoextra (https://cloud.r-project.org/package=factoextra) to show the clustering of samples with the first two components. After normalizing the reads by TPM (Tags Per Million) of each gene in samples, in house-script (sogen) was used for visualization of next-generation sequence data and genomic annotations. The pheatmap package (https://cran.r-project.org/web/packages/pheatmap/index.html) in R was used to perform the clustering based on Euclidean distance.

### Animal grouping and modelling

Twelve male Sprague-Dawley (SD) rats (8 weeks old) were purchased from Beijing Huafukang Biological Technology Co., Ltd., China (No. 110,322,251,103,728,758). All animal experiments were conducted in accordance with the regulations of the Institutional Animal Care and Use Committee (IACUC) of Shouzheng Pharma (Wuhan) Biotechnology Co., Ltd. (Approval number: 2,025,120,401), and all procedures complied with relevant institutional guidelines for animal care and use. Rats were housed under standard laboratory conditions (22 ± 2°C, 12 h light/dark cycle) with free access to food and water. The sample size was determined based on previous similar studies and preliminary experimental experience. The rats were randomly divided into three groups (*n* = 4 per group): the sham group, the CCI model group, and the CCI + l-THP group (l-THP, No. HY-N0096, MCE, USA, 64 mg/kg/day). The chronic constriction injury (CCI) model was established as follows [10]. After the rats were anesthetized with 1% sodium pentobarbital, the left sciatic nerve was exposed and loosely ligated with 4–0 catgut at approximately 1 mm intervals. In the sham group, the sciatic nerve was exposed and isolated without ligation. The muscle and skin were sutured layer by layer and sterilized with iodine. From postoperative day 6 to day 10, rats in the CCI + L-THP group received daily intragastric administration of 64 mg/kg L-THP, while the sham and CCI groups received an equal volume of 0.5% CMC-Na. Exclusion criteria were predefined before the study. Animals with severe postoperative complications or accidental injury unrelated to the experimental intervention were to be excluded. Samples were excluded only in cases of clear technical failure during tissue processing or molecular analysis. No animals or samples met these criteria.

### Pain behavioural assessments

Behavioural tests, including mechanical withdrawal threshold (MWT) and thermal withdrawal latency (TWL), were measured in the three groups at baseline (day 0, before surgery) and on postoperative days 1, 5 and 9. To evaluate mechanical sensitivity, the mechanical withdrawal threshold was measured using the von Frey filaments (Exacta Touch-Test^TM^ Sensory Evaluators) to stimulate the centre of the injury side paws. Animals were placed in a plexiglass cage with a wire mesh floor and allowed to acclimate. Calibrated von Frey filaments were applied perpendicularly to the plantar surface of the hind paw. The withdrawal threshold was recorded [11]. Thermal Hyperalgesia Test was assessed using the Intelligent Hot Plate Instrument (SA705, Jiangsu SansBio Biotechnology Co, China). The thermal withdrawal latency was measured by directing a focused heat source to the plantar surface of the hind paw until the animal exhibited a withdrawal response [12]. Behavioural assessments, histological evaluation and data analysis were performed by investigators blinded to group allocation.

### Immunohistochemistry

Lumbar spinal cord tissue sections were placed into an antigen retrieval buffer and heated in a microwave at a medium power for 15–20 minutes and rinsed in PBS buffer three times (5 minutes each). To block endogenous peroxidase activity, after being rinsed with phosphate buffered saline, the sections were incubated in 3% Methyl alcohol-H_2_O_2_ solution for 25 minutes. The sections were rinsed three times (3 minutes each) in phosphate buffered saline and incubated in a 3% BSA blocking solution (goat serum in phosphate buffered saline) for 30 minutes at 20°C. The sections were incubated overnight with primary anti-Grn/Actn3 antibodies at 4°C. The antibodies used were listed in Supplementary Table S1. After three washes with phosphate buffered saline, the sections were incubated with HRP-conjugated secondary antibody (S-vision IHC Polymer, goat anti-rabbit, G1302, Servicebio, China) for 50 minutes at 20°C. Finally, immunodetection was achieved using the DAB staining solution. After a series of dehydration steps using xylene and ethyl alcohol, the sections were mounted onto slides [13]. The sections were visualized under an inverted microscope (E100, Nikon, Japan).

### Immunofluorescence staining

The 25-μm-thick SC tissue sections were washed with PBS and incubated with 0.2% Triton X-100 + 10% goat serum for 1 h at room temperature. Subsequently, the sections were incubated overnight with appropriate primary antibodies at 4°C and incubated with fluorescent-labelled secondary antibodies on the next day for 2 h. The nucleus was stained using DAPI (C1005, Beyotime) for 8 min [14]. Finally, the sections were scanned via a confocal microscope (Nikon Eclipse C1, Japan). Dried, coverslipped IF slides were scanned with Pannoramic Midi (3DHistech, Budapeste). The antibodies used were listed in Supplementary Table S1.

### Junction-specific qRT-PCR validation of as events

In this study, to elucidate the validity of the RNA-seq data, RT-PCR was performed for some selected AS events. The PCR conditions are consisted of denaturing at 95°C for 5 min, 40 cycles of denaturing at 95°C for 10 seconds, annealing and extension at 60°C for 30 seconds. PCR amplifications were performed in triplicate for each sample. The primers used for junction-specific qRT-PCR assay were designed in junction reads and adjacent exon to ensure specific amplification of spliced RNA (AS events) and unspliced RNA (Model events) [15]. Primers for qPCR analysis were listed in Table S2.

### Other statistical analysis

Data was analysed using GraphPad Prism 8.0 (San Diego, USA). Data was analysed using one-way ANOVA with Tukey’s post hoc test for multiple groups comparisons. Time-series data were analysed with the two-way ANOVA. *p* < 0.05 was considered statistically significant.

## Results

### Part 1. Identification and characterization of alternative splicing events in spinal cord samples from a chronic constrictive injury rat model

**Figure 1.**
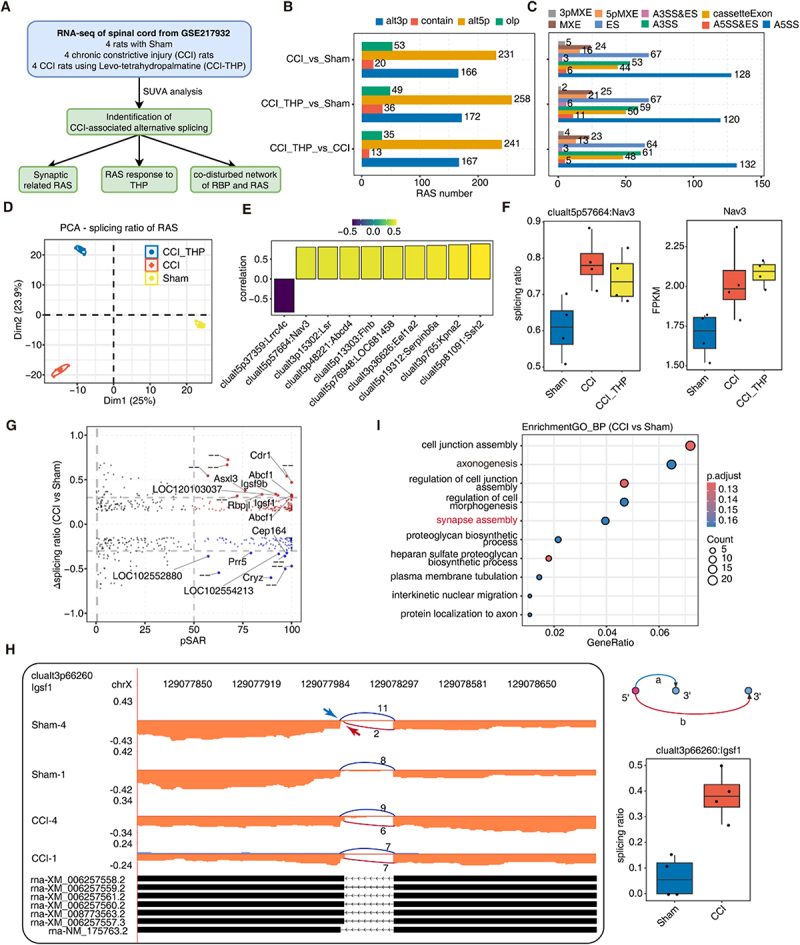
Identification and characterization of alternative splicing events in spinal cord samples from a chronic constrictive injury rat model. A. Workflow of bioinformatic analysis in this study. B. Bar plot showing number of regulated alternative splicing events (RAS) among Sham, CCI and CCI_THP samples detected by SUVA. C. Splice junction constituting RAS events detected by SUVA was annotated to classical AS event types. And the number of each classical AS event types were showed with bar plot. D. Principal component analysis (PCA) of splicing ratio of RAS. The samples were grouped by PNALD or normal and the ellipse for each group is the confidence ellipse. Ranked bar plot illustrating the correlation between the splicing ratio of RAS and the expression of its host gene. Correlations with |Pearson’s correlation coefficient| ≥0.8 and *p*-value ≤0.01 are displayed. E.Boxplot showing splicing ratio of RAS clualt5p57664: Nav3 and expression profile of its hostgene in Sham, CCI and CCI_THP samples. F. Scatter plot depicting the difference in splicing ratio and the median pSAR of each RAS when comparing CCI and Sham samples. In the plot, red and blue points represent RAS with pSAR >50%. Gene names are labelled for RAS with Δsplicing ratio ≥0.3 or ≤−0.3, according to the applied cut-offs. G. Visualization of reads distribution of Igsf1 in AS events clualt3p66260 from different groups. Splice junctions were labelled with SJ reads number. And altered splice sites were marked out with arrow. Boxplot in the right panel showing splicing ratio profile of CCI and Sham samples of the splicing event from Igsf1 showed in left panel. H. The top 10 most enriched Gene Ontology (GO) terms related to biological processes were visualized for genes involved RAS when comparing CCI and Sham samples.

To investigate the key patterns of alternative splicing regulation in a chronic nerve injury model more comprehensively, we utilized the publicly available transcriptome dataset GSE217932, which comprises spinal cord tissues from rats experiencing neuropathic pain following chronic nerve injury. This study involved transcriptomic analysis of 12 samples, including four from the sham-operated group (Sham), four from the chronic constriction injury group (CCI) and four from the CCI treated with L-Tetrahydropalmatine (CCI_THP). We performed rigorous quality control and comprehensive analysis on these data, and subsequently analysed focusing on the differential alternative splicing events, particularly those involving genes associated with synaptic remodelling. Additionally, we constructed a regulatory network linking RNA-binding proteins and alternative splicing events in neurons. The overall technical workflow is depicted in (Figure 1(A)). Using SUVA software [10], alternative splicing events were classified into four categories: alternative 3’ splice site (alt3p), alternative 5’ splice site (alt5p), overlapping (olp) and contain events (Figure S1A). The SUVA analysis identified tens of thousands of splicing events in each sample group (Figure S1B). The types of alternative splicing events that varied between the three groups were subsequently quantified. The primary differential alternative splicing event types identified were alt5p and alt3p events (Figure 1(B)). Corresponding SUVA event types to classical alternative splicing events, the predominant alternative splicing event type after CCI injury was alternative 5’splicing A5SS, followed by exon jump ES events, and alternative 3’ splicing A3SS (Figure 1(C)). Approximately two-thirds of the significantly distance alternative splicing events observed across the three group comparisons were classified as complex splicing events. Complex splicing events are defined as splicing sites that are utilized by one or more types of alternative splicing events (Figure S1C). These findings highlight the complex nature of alternative splicing events in spinal cord tissues. Principal component analysis (PCA) demonstrated a significant separation among the three groups (Figure 1(D)), suggesting that the alternative splicing landscapes of these sample groups underwent substantial changes.

It is considered that alternative splicing may affect sense-mediated mRNA degradation, thereby impacting gene expression. Subsequently, we analysed the correlation between the four types of splicing events identified by splicing ratio and the expression levels of their corresponding host genes. We identified genes and splicing events that exhibited significant correlations (|correlation coefficient|≥0.8, *p*-value ≤0.01). Notably, the gene Lrrc4c demonstrated a significant negative correlation with its expression post-splicing. In contrast, the genes Nav3, Lsr, Abcd4, Flnb, Eef1a2, Serpinb6a, Kpna2 and Ssh2 exhibited a positive correlation between the splicing event ratio and gene expression (Figure 1(E)). This suggests that certain genes expression pattern may be influenced by alternative splicing events. For instance, the gene Nav3 undergoes alternative splicing at the 5’ end (alt5p) following CCI injury, resulting in an increase splicing ration compared to the sham operation group, and an upregulation of gene expression post-CCI injury (Figure 1(F)). Moreover, the Flnb gene exhibits a similar trend, with the alt5p event increasing following CCI injury and subsequently decreasing after THP treatment (Figure S1D-E). The gene Flnb encodes a protein belonging to the filament protein family, which interacts with cytoskeletal actin to facilitate the anchoring of various actin-associated proteins to the cytoskeleton. It has been shown that Flnb plays a role in the development of cortical neurons in the brain [16]. However, the involvement of Flnb in neuropathic pain remains to be elucidated.

The results indicate that CCI injury leads to complex splicing regulation in spinal cord tissues. We identify the most significant splicing changes affecting gene-dominant transcripts after CCI injury, involving genes like Igsf1, Rbpjl, Abcf1, Igsf9b, Asxl3, Cdr1 (Figure 1(G)). (Figure 1(H)) demonstrates the alt3p alternative splicing events occurring in the gene Igsf1, with a significant increase in the selection of proximal 3’ splice sites in the nerve pain CCI group compared to the Sham group. The gene Igsf1 a member of the Immunoglobulin superfamily, is predominantly expressed in hypothalamic and pituitary cells. Mutations in this gene in humans are associated with conditions such as hypothyroidism, megatestosteronism and others [17]. We discovered that the Atm gene, linked to neuroinflammatory, exhibits splicing changes after CCI injury (Figure S1F). GO-BP enrichment analysis revealed that genes with alternative splicing post-CCI are primarily enriched in pathways related to neuronal function and development, such as cellular junction assembly, axon generation, cellular morphology regulation and synaptic assembly (Figure 1(I)). This suggests significant post-transcriptional regulation in neural recovery and synaptic remodelling after CCI injury. Comparing the GO-enriched pathways associated with differential alternative splicing events across the three treatment groups, it was observed that pathways related to axon generation were enriched in all groups. Notably, pathways associated with RNA splicing regulation exhibited significant enrichment following THP treatment compared to the CCI nerve pain group (Figure S1G).

### Part 2. Alternative splicing of genes associated with synaptic formation and remodelling undergoes significant regulation during the onset of neuropathic pain.

**Figure 2.**
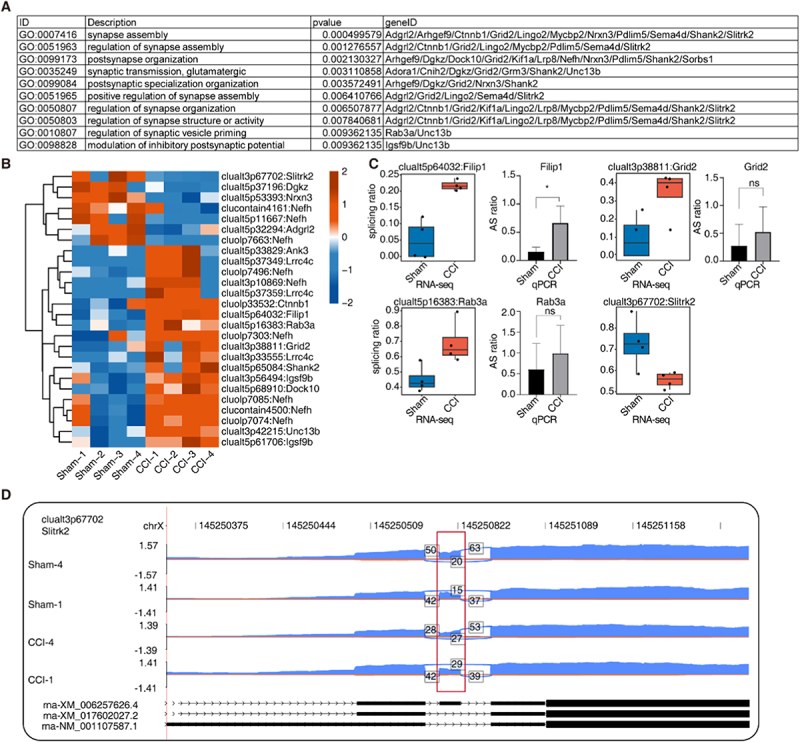
Alternative splicing of genes associated with synaptic formation and remodeling undergoes significant regulation during the onset of neuropathic pain. A. Table displaying the most enriched pathways associated with synaptic plasticity for genes involving RAS when comparing CCI and Sham samples B. Heatmap of splicing ratio levels (z score, row scaled) across Sham and CCI samples for synaptic plasticity-related RAS. C. Validation of RAS by junction-specific qRT-PCR analysis. Boxplot showing expression profile of four synaptic plasticity-related RAS in Sham and CCI samples. Junction-specific qRT-PCR analysis is performed to detect the change in AS ratio (Ratio = AS splicing/(AS splicing + model splicing)). β-actin is used as endogenous control. Error bars represent the mean ± SD from three independent experiments. *p < 0.05. ns *p* ≥ 0.05. D. Visualization of reads distribution of Slitrk2 in AS events clualt3p67702 from different groups. Splice junctions were labelled with SJ reads number. And altered exon was marked out with box. Boxplot in the right panel showing splicing ratio profile of Sham and CCI samples of the splicing event from Slitrk2 showed in left panel.

Synaptic remodelling is a critical component in the pathophysiology of pain following nerve injury. Our study identified significant alterations in the patterns of alternative splicing regulation of numerous genes associated with synapse formation and synaptic remodelling in both chronic constriction injury (CCI) and sham-operated groups. The genes undergoing these alternative splicing events were found to be enriched in pathways related to synaptic assembly, postsynaptic organization, synaptic transmission and other related pathways (Figure 2(A)). In the CCI group, there was an observed increase in the alternative splicing events of the screened genes Lrrc4c, Nefh, Ctnnb1, Filip1, Rab3a and Grid2, whereas a decrease in the alternative splicing events was noted for Slitrk2 and Nrxn3. To verify the abnormal splicing events caused by CCI, we constructed a CCI rat model and a CCI model treated with THP as per the experimental paper by Wu DX et al. The von Frey mechanical withdrawal threshold (PWT) demonstrated an increase from 4.1 g ± 0.9 g in chronic constriction injury (CCI) model rats treated with vehicle CMC-Na^+^ to 7.6 g ± 2.5 g in rats treated with L-tetrahydropalmatine (L-THP). Similarly, the thermal withdrawal latency (PWL) improved markedly from 5.47 ± 1.02 seconds in CCI rats to 10.36 ± 1.0 seconds in L-THP-treated rats (*p* < 0.001), indicating a significant reduction in thermal hyperalgesia. The behavioural results showed that THP could significantly alleviate the thermal pain threshold of CCI rats (Figure S2A-B). Among the various alternative splicing events analysed, four were selected for validation by junction-specific qPCR. The splicing patterns of three events (Filip1, Grid2 and Rab3a) were consistent with the RNA-seq data. However, primer design for validating the Slitrk2-associated splicing event was not feasible due to the insufficient length of its target sequence. qPCR primers summarized in supplementary Table 2 (Figure 2(B-C)). The axon-specific guidance molecule Lrrc4c, whose deletion is associated with neurodevelopmental disorders [18]. Gene Nefh functions as a serum biomarker for degenerative diseases of motor neuron [19]. Ctnnb1 has been reported to exhibit up-regulated expression in the brain under conditions of neuropathic pain, functioning as a transcription factor potentially involved in the regulation of cell adhesion and receptor activity [20]. The expression of Ctnnb1 was also observed to be up-regulated in the dorsal root ganglion tissues of rats with nerve-injured [21]. Filip1 may play a role in the regulation of spinal neuromorphology, and the knockdown of Filip in murine models has been shown to impact the transmission of neural excitation [22]. The gene Rab3a plays a crucial role in the regulation of synaptic vesicles, influencing their transit to the active zone and subsequent docking [23]. Grid2, also known as GluD2, is involved in synapse formation [24]. Slitrk2 is responsible for regulating synaptic growth and excitatory transmission, knockout studies in mice have demonstrated that the absence of Slitrk2 leads to impaired long-term memory and synaptic excitatory function, culminating in neurodevelopmental deficits [25]. The neurexin gene Nrx3 function as a synaptic adhesion molecule, with mutations in this gene being linked to autism, schizophrenia and Alzheimer’s disease [26]. The aforementioned synapse-associated genes were examined using the protein–protein interaction (PPI) network to identify potential interactions, such as those between Slitrk2 and Nrxn3 (Figure S2C). Notably, the reads distribution map of Slitrk2 indicated a decreased retention ratio of a specific exon in the CCI group (Figure 2(D)). Conversely, the reads distribution plot of the gene Grid2 demonstrated an increased retention of a particular exon in the CCI group (Figure S2D).

### Part 3. Levo-tetrahydropalmatine treatment restores aberrant splicing patterns of synaptic remodelling-related genes.

**Figure 3.**
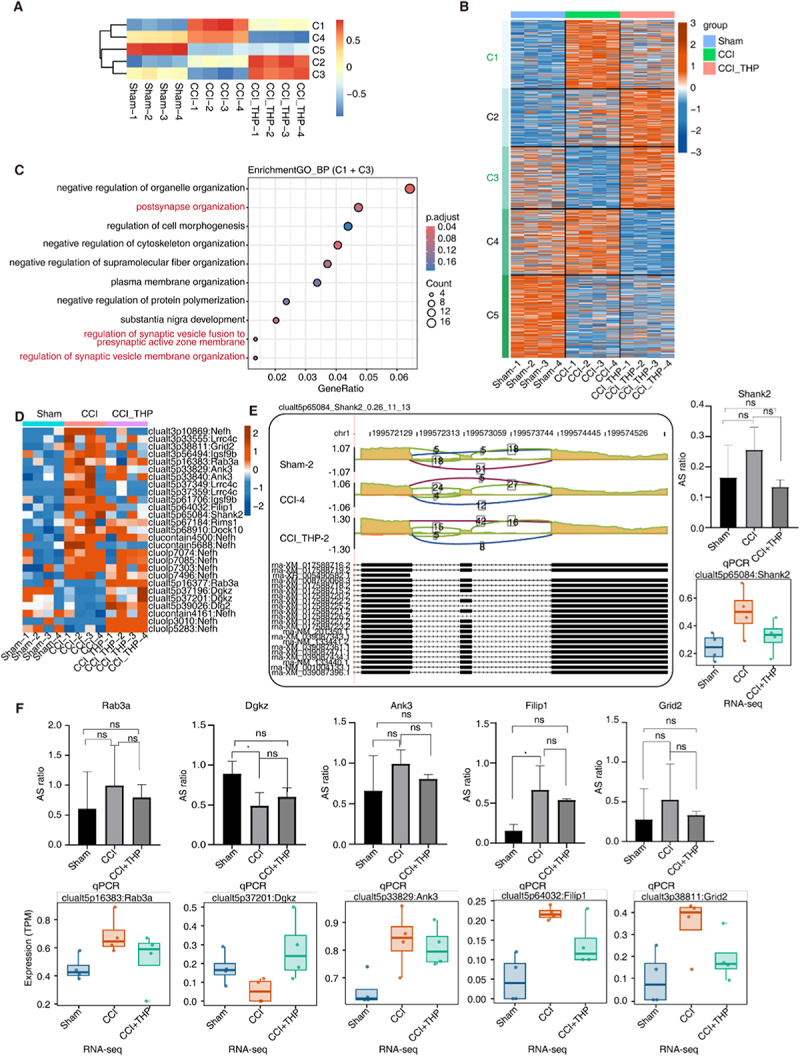
Levo-tetrahydropalmatine treatment restores aberrant splicing patterns of synaptic remodeling-related genes. A–B. RAS among Sham, CCI and CCI_THP were clustered using K-means. Heatmap in up-panel presenting 5 clusters of RAS identified by K-means clustering and heatmap B showing splicing ratio patterns according clustered RAS. A. The top 10 most enriched Gene Ontology (GO) terms related to biological processes were visualized for genes involved RAS from C1 and C3 clusters. B. Heatmap of splicing ratio levels (z score, row scaled) across all samples for synaptic plasticity-related RAS from C1 and C3 clusters. C. Visualization of reads distribution of Shank2 in AS events clualt5p65084 from different groups. Splice junctions were labelled with SJ reads number. And altered exon was marked out with box. Boxplot in the right panel (down) showing splicing ratio profile of Sham, CCI and CCI_THP samples from RNA sequencing data of Shank2. Validation of alternative splicing events by qRT-PCR (up panel). D. Junction-specific qPCR validation of alternative splicing ratios for *Rab3a, Dgkz, Ank3, Filip1* and *Grid2*. Data are presented as mean ± SD; * *p*<0.05. ns, no significant.

L-tetrahydropalmatine (THP) has been extensively utilized for the alleviation of both acute and chronic pain [27]. However, the precise mechanisms by which it acts against neuropathic pain remain inadequately. We hypothesized that THP might exert post-transcriptional modulatory effects by restoring aberrant splicing regulation. To investigate the patterns of alternative splicing regulation across the three treatment groups, we conducted k-means clustering analysis on the splicing ratios of all differential splicing events. The analysis revealed that these splicing events could be significantly clustered into five distinct clusters (C1-C5), with the genes within each cluster being enriched in various aspects of nervous system function (Figure S3A). Notably among them C1 exhibited an increased splicing event ratio in the CCI group. However, treatment with THP resulted in a reduction of this ratio, aligning it more closely with that observed in the Sham group. Conversely, C3 demonstrated a decreased splicing event ration in the CCI group, but this ratio increased following THP treatment, mirroring the trend seen in the Sham group (Figure 3(A-B)). Alternative splicing events in clusters C1 and C3 may be influenced by THP treatment, potentially restoring the dysregulated splicing patterns associated with CCI injury. Genes within C1 and C3 are predominantly enriched in pathways related to organelle organization, synaptic function and the regulation of cell morphology regulation (Figure 3(C)). We generated a heat map illustrating the splicing event ratios of genes associated with synaptic function in C1 and C3, revealing that 27 splicing events related to synaptic remodelling exhibited restoration of normal splicing patterns following THP treatment (Figure 3(D)). Reads distribution maps highlighted the complex splicing events in the Shank2 gene, with significant differences in the ratio of the two exon-skipping splicing phenotypes between the Sham group to the CCI group. Notably, the THP treatment group demonstrated a return to the splicing pattern observed in the Sham group (Figure 3(E)). Shank2 is a multi-domain scaffolding protein involved in the structural and functional coordination of multi-protein complexes at excitatory postsynaptic sites and has been associated with psychiatric disorders, including autism spectrum disorders [28–30]. To verify the impact of THP treatment on aberrant splicing events associated with synaptic remodelling, a junction-specific qPCR assay was employed to examine the variable splicing events of the Shank2 gene. While the results did not reach statistical significance, observations indicated that the CCI model facilitated the exon-skipping splicing pattern, which was subsequently reversed by THP treatment. Furthermore, the study extended to evaluate the splicing patterns of additional genes, including *Rab3a, Dgkz, Ank3, Filip1*, and *Grid2*. While these splicing events also did not achieve statistical significance, the observed trends were consistent with RNA-seq sequencing data (Figure 3(F)). Consequently, the therapeutic effects of THP may also manifest in the correction of aberrant splicing events during synaptic remodelling.

### Part 4. Construction of a co-regulatory network for RBPs and RAS events reveals potential mechanisms in neuropathic pain

**Figure 4.**
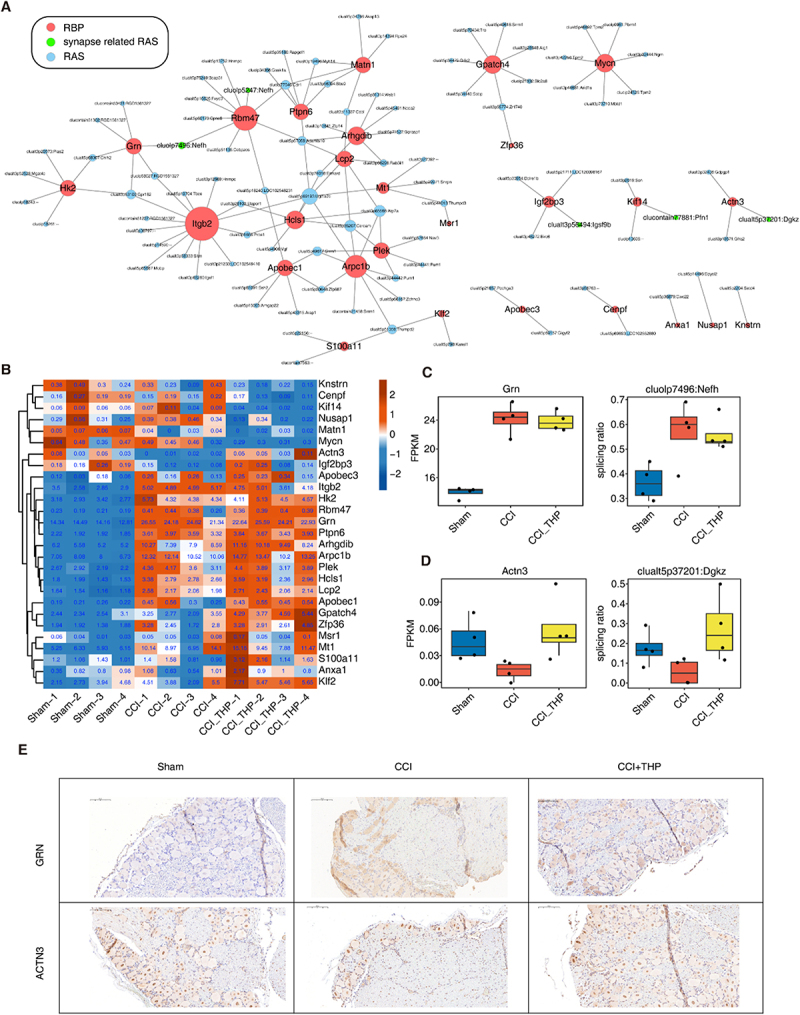
Construction of a co-regulatory network for RBPs and RAS events reveals potential mechanisms in neuropathic pain. A. The co-disturbed network among expression of DE RBPs and splicing ratio of CCI-RAS events. |Pearson’s correlation| ≥0.85 and *p* value ≤0.01 were retained for RBP and RAS correlation. The red circles denote RBPs and other circles denote RAS. Green circles denote synaptic plasticity-related RAS. B. The heatmap diagram showing the expression profile of DE RBPs involved in the co-disturbed network from A. C. Boxplot showing expression profile of RBP Grn and targeted RAS cluolp7496: Nefh in Sham, CCI and CCI_THP samples. D. Boxplot showing expression profile of RBP Actn3 and targeted RAS clualt5p37201: Dgkz in Sham, CCI and CCI_THP samples. E.Immunohistochemical analysis of GRN and ACTN3 protein expression in DRG tissues. Scale bars =100 μm.

To thoroughly examine the regulatory mechanisms of alternative splicing in the context of neuropathic pain development, particularly given its primary regulation by RNA-binding proteins, a correlation analysis was conducted. This analysis focused on differential alternative splicing events and differentially expressed RNA-binding proteins across three treatment groups, utilizing Pearson’s correlation analysis. Initially, the differentially expressed genes within these groups were identified, yielding a total of 1259 differentially expressed genes (Figure S4A). Among these, 27 RNA-binding proteins exhibited differential expression regulation (Figure S4B). We examined the correlation between the splicing ratios of all differential alternative splicing events and the expression levels of differential RNA-binding protein (RBP) genes. By applying a filtering criterion of |Pearson’s correlation coefficient|≥0.85, *p*-value ≤0.01, we constructed a network to explore the potential regulatory interactions between RBP genes and RAS events (Figure 4(A)). Our analysis revealed a significant association between the alternative splicing events of gene Nefh, which is implicated in motor neuron degenerative disease, and the expression of splicing factors Rbm47 and Grn. Furthermore, the expression heatmap indicated that the expression levels of Rbm47, Grn, Lcp2, Plek, Ptpn6 and Hcls1 were significantly elevated in the CCI-treated group (Figure 4(B) and Figure S4B). Notably, the expression trend of Grn was consistent with the splicing trend of Nefh (Figure 4(C)), while the splicing trend of Actn3 aligned with the expression trend of Dgkz (Figure 4(D)). The Grn gene encodes a protein referred to as forebrain neuronal cell-specific glycoprotein. Investigations into Grn proteins have demonstrated their significant roles within the nervous system, particularly in relation to vestibular neurons and microglia, including astrocytes. Research indicates that Grn proteins may contribute substantially to neuroprotection, neuronal survival and the pathophysiology of neurodegenerative diseases. Mutations or deletions within specific regions of the Grn gene may be implicated in neurological disorders such as vestibular neuron disease and frontotemporal dementia [31,32]. This indicated the RBPs and RAS events are all contributed in neuropathic pain.

Consistent with the transcriptional changes, immunohistochemical staining (Figure 4(E)) revealed dysregulation of GRN and ACTN3 in the dorsal root ganglion (DRG) of CCI rats. Treatment with THP mitigated the CCI-induced overexpression of GRN, while it facilitated the upregulation of ACTN3 protein levels. Collectively, these results indicate that THP modulates the expression of key pain-related RBPs.

### Part 5. Spatial correlation of Grn, Nefh, Actn3 and Dgkz

**Figure 5.**
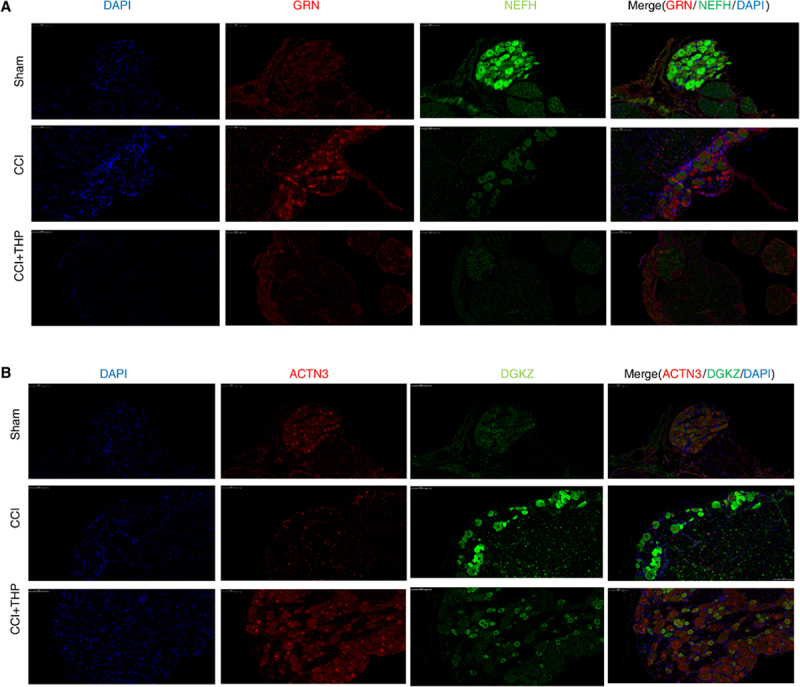
Altered levels and localization of RNA-biding proteins and RAS genes in response to THP treatment in CCI rats. A. Representative confocal images show DAPI (blue, nuclei), GRN (red), NEFH (green) and merged channels in sham, CCI and CCI+THP groups. Orange signals in merged images indicated co-localization. Scale bar = 100 μm. B. Representative confocal images show DAPI (blue, nuclei), ACTN3 (red), DGKZ (green) and merged channels in sham, CCI and CCI+THP groups. Orange signals in merged images indicated co-localization. Scale bar = 100 μm.

Building on previous analyses and experimental findings, it has been demonstrated that THP treatment significantly affects the expression of key RBP genes and the alternative splicing of genes involved in synaptic remodelling. To further verify the spatial relationship between RBPs and RAS-related genes in DRG neurons following neuropathic injury and THP treatment, immunofluorescent co-staining for GRN, NEFH, ACTN3 and DGKZ were conducted in sham, CCI and CCI+THP groups. In the sham groups, the levels of GRN were low, with limited co-localization signals observed with NEFH. Conversely, in the CCI group, GRN expression was upregulated, and the merged imaging distinctly demonstrated co-localization of GRN and NEFH within the cell nucleus. Following THP intervention, there was a downregulation of GRN expression, accompanied by a significant reduction in the co-localization of GRN and NEFH (Figure 5(A)). In the sham group, ACTN3 and DGKZ exhibited low basal expression with mild co-localization in DRG neurons. CCI-induced neuropathic injury triggered a marked upregulation of DGKZ, with co-localization with ACTN3 in cell nucleus, accompanied by diffuse ACTN3 distribution. Notably, THP administration significantly reversed the CCI-induced overexpression of DGKZ, while further upregulating ACTN3 expression, and normalized their co-localization pattern in DRG neurons (Figure 5(B)). In summary, the findings of this study suggest that THP mitigates neuropathic pain through the modulation of aberrant GRN and ACTN3 expression in dorsal root ganglion neurons. This regulation appears to affect the alternative splicing events related to these RBPs, ultimately leading to a reduction in neuronal damage and synaptic dysfunction.

## Discussion

We conducted an alternative splicing analysis on RNA-seq data derived from spinal cord tissue subjected to chronic nerve injury. Additionally, we carried out a genome-wide assessment of aberrant alternative splicing events in spinal cord tissue associated with initial neuropathic pain, utilizing SUVA process. Notably, the alternative splicing events of certain genes were found to influence some gene expression. Among these, the axon-specific guidance molecule Lrrc4c, whose deletion mutation is implicated in neurodevelopmental disorders [18]. The gene Nav3 (also known as UNC-53), which plays a role in nerve growth and regeneration, was particularly significant [33]. These findings indicate that chronic nerve injury induces aberrant regulation of alternative splicing in genes associated with nerve development and regeneration within spinal cord tissue, accompanied by alterations in their expression levels. A comprehensive GO enrichment analysis of genes involved in alternative splicing events across the three treatment groups revealed significant enrichment in pathways related to axonogenesis and synaptic remodelling. Prior studies have shown that axonogenesis [34] and synaptic remodelling [35] are the main pathways leading to chronic neuropathic pain. Furthermore, peripheral nerves possess the capacity for regeneration, and active nerve regeneration is crucial for the persistence of neuropathic pain.

Regenerated neurons serve as the primary source for detecting abnormal activity within the body, and interference with neuronal regeneration may alleviate pain. Our study also examined the enrichment of synaptic remodelling-related pathways in the CCI group. Previous research has identified synaptic remodelling as a key pathway contributing to chronic neuropathic pain [3,36]. In our analysis, we concentrated on the alternative splicing of genes associated with the synaptic remodelling pathway. This finding revealed that the genes such as Lrrc4c, Ctnnb1, Filip1, Rab3a, Grid2, Slitrk2 and Nrx3, which are involved in regulating synaptic growth, exhibited abnormal splicing in the CCI group. To directly validate our computational predictions, a series of validation experiments in the CCI rat model were conducted. Behavioural assays confirmed that L-THP treatment alleviated both mechanical allodynia (von Frey PWT) and thermal hyperalgesia (Hargreaves PWL), establishing the functional relevance of our treatment paradigm. At the molecular level, junction-specific RT-qPCR analysis of six computationally predicted splicing events of genes *Dgkz*, *Filip1*, *Grid2*, *Rab3a*, *Ank3* and *Shank2*, which revealed expression patterns coincident with our bioinformatic predictions: all six alternative splicing events showed dysregulation in CCI animals and were normalized by L-THP treatment.

This multi-gene concordance substantially reduces the probability that our computational findings represent false-positive predictions and provides the first in vivo mRNA-level evidence that these AS events are pharmacologically modifiable. The six validated genes converge on two crucial roles of central sensitization: regulation of excitatory synaptic strength, and modulation of intrinsic neuronal excitability. ***Shank2*** encodes a scaffold protein to link NMDA receptors with GKAP and SAPAP during synaptic plasticity [37]. Its splice isoforms differentially anchor NMDA receptor [38], which is involved in dorsal horn sensitization and pain [39]. Disruption of *Shank2* splicing patterns could alter the distribution of glutamate receptors at sensitized synapses. ***Grid2*** encodes the delta-2 ionotropic glutamate receptor subunit (GluD2). Blocking the N-terminal domain of Grid2 can abolish synaptic plasticity [40]. The ***Dgkz*** gene encodes diacylglycerol kinase zeta (DGKζ), an enzyme involved in the regulation of excitatory synaptic transmission. DGKζ facilitates the transmission of excitatory synaptic by phosphorylating diacylglycerol (DAG) to form phosphatidic acid [41]. Aberrant splicing of *Dgkz* in the CCI rats may consequently influence synaptic transmission. ***Rab3a***, a small GTPase critical for synaptic vesicle secretion and transport [42], is significantly downregulated in spinal cord injury rats and inhibits M1 macrophage polarization through neuronal-derived vesicle secretion [43]. **Ank3** organizes the axon initial segment and nodes of Ranvier [44,45]. An aberrant isoform of *Ank3*, E35a enriched in GABAergic neurons, controls interneuron excitability and intracellular Ca^2+^ signalling via InsP3Rs interactions [46]. Aberrant *Ank3* splicing in the CCI spinal cord may contribute to excessive interneuron excitability [46]. ***Filip1*** is required for proper neural spine morphology, and its loss-of-function leads to abnormal excitation propagation through disruption of the accumulation of NMDA [22]. These six genes encompass the entire spectrum of synaptic function, ranging from presynaptic release (*Rab3a*) through receptor organization (*Grid2*, *Shank2, Dgkz*) to neural morphology remodelling (*Filip1*) and excitability regulation (*Ank3*). The aberrant splicing regulation observed in CCI and normalization followed by L-THP treatment, suggests that L-THP mitigates neuropathic pain by modulating alternative splicing programs.

In addition to the validation of alternative splicing events, immunohistochemical analysis of ACTN3 and GRN proteins further corroborated the transcript-level findings. This analysis demonstrated that L-THP treatment influences protein-level changes in key RBP genes within the lumbar spinal cord.

Integrating our animal experiments, bioinformatics analysis, we propose a multi-level mechanistic model in which L-THP suppresses neuroinflammatory signalling, restores RBP expression and thereby recalibrates the alternative splicing landscape towards a homoeostatic state.

Furthermore, the original study demonstrated that treatment with THP resulted in the downregulation of Clec7a expression and decreased phosphorylation levels of MAPK and NF-kB-p65 induced by Clec7a, leading to a reduction in the expression of pyroptosis-related proteins NLRP3 and Caspase-1-p20 [10]. This project identified that THP [47,48] may alleviate pain by modulating abnormal alternative splicing. Mutations in the gene Grn have been implicated in neurodegenerative diseases, with evidence suggesting that Grn expression is upregulated in activated microglia, thereby influencing neuroinflammation. However, there is a paucity of research on its regulatory function as an RNA-binding protein. The results of this analysis demonstrate a correlation between Grn expression RNA-binding protein and the regulation of alternative splicing of the Nefh gene.

Previous studies have identified Nefh as a potential serum biomarker for motor neuron degenerative diseases [49]. In this study, aberrant splicing of Nefh was observed in pathological tissues associated with neuropathic pain. The Dgkz (Diacylglycerol kinase zeta) gene encodes the Dgkz protein, which is crucial in neurons for regulating neural signal transmission. The primary function of Dgkz is mediated through the regulation of diacylglycerol (DAG) levels, a key signalling molecule involved in multiple cellular signalling pathways, particularly in synaptic plasticity [50]. Synaptic remodelling involves the coordinated action of multiple molecules and signalling pathways. As an enzyme implicated in membrane lipid metabolism, Dgkz is posited to have a significant role in synaptic plasticity [51]. Research indicates that synaptic remodelling is contingent not only upon structural modification within synapses but also upon the regulation of neural activity and optimization of signal transmission [52]. Consequently, Dgkz may modulate synaptic activity and functionality by regulating DAG levels, thereby contributing to the synaptic remodelling process.

Several limitations of the present study warrant discussion. First, our transcriptomic analysis is based on a single publicly available dataset (GSE217932) derived from lumbar spinal cord (L4–L6) of CCI rats at 10 days post-surgery, representing one model, one CNS compartment and one time point along the neuropathic pain trajectory. It remains to be determined whether the alternative splicing events identified in this study are conserved across other commonly utilized neuropathic pain models, such as the spared nerve injury (SNI) model, the complete Freund’s adjuvant (CFA) model, or the spinal nerve ligation (SPL) model, or if they persist at later chronic time points (beyond Day 28). A transcriptomic analysis of alternative splicing by Zhai et al. [53] identified a shared set of alternatively spliced genes across CFA and SNI models in the spinal cord, suggesting that some conserved AS events may represent common features of chronic neuropathic pain. However, direct AS-level cross-model comparisons are needed to confirm this. Second, our laboratory’s verification adopted the same CCI model and tissue types as the original dataset. Although this does not solve the limitations of a single model, it has increased the sample size. Future studies should examine AS dynamics in DRG, brain regions involved in pain processing (e.g. cortex, thalamus), and in alternative pain models. Third, while our data show co-localization between ACTN3, GRN protein levels and the expression of its predicted target gene DGKZ and NEFH, we have not yet established a causal relationship between RBPs and their targets through gain- or loss-of-function experiments. Knockdown or overexpression of RBPs genes in vivo will be required to formally test whether this factor is necessary and sufficient to drive the observed AS changes.

## Conclusion

In summary, this study presents a comprehensive transcriptome-wide analysis of alternative splicing (AS) in the lumbar spinal cord of a CCI rat model of neuropathic pain and its pharmacological reversal by L-THP. We have revealed hundreds of credible and dominant RAS events enriched in pathways related to nerve cell function and neural development, such as cell junction assembly, axonogenesis, regulation of cell morphology and synapse assembly. AS events were associated with the regulation of synaptic formation and remodelling undergoes significant regulation during the onset of neuropathic pain. There is a co-alteration regulated network involved in RAS and RBPs. Rbm47, Grn, Lcp2, Plek, Ptpn6, Hcls1, Nefh, Actn3 and Dgkz may serve as molecular targets for neuropathic pain. Six alternative splicing events — *Dgkz*, *Filip1*, *Grid2*, *Rab3a*, *Ank3* and *Shank2* — were independently validated by RT-qPCR, and two RBP proteins (ACTN3, GRN) were confirmed by immunohistochemistry, collectively supporting a multilevel synaptic vulnerability model of neuropathic sensitization. This may provide new therapeutic strategies or molecular targets for the treatment of neuropathic pain in the future. Despite conducting comprehensive analyses in this study, certain limitations remain, including a small sample size and the absence of validation using clinical samples. Functional verification is planned for subsequent research.

## Supplementary Material

Supplemental Material

Supplemental Material

## Data Availability

The datasets used and/or analysed during the current study are available under the gene expression omnibus series accession number GSE217932. Any further underlying data will be made available upon reasonable request.
