# Supplementary material for: Comprehensive profiling and molecular characterization of alternative splicing regulation in synaptic remodelling associated with neuropathic pain induced by chronic constrictive injury in a rat model
Source: RNA Biol. 2026 May 15;23(1):1–18. doi: 10.1080/15476286.2026.2675080 (PMC13240960; doi:10.1080/15476286.2026.2675080)
Supplement: Supplemental Material [file KRNB_A_2675080_SM3595.pdf]

Supplementary Table 1. The information of antibodies used in this study.

Antibody	Cat. No.	Dilution	Brand
GRN	18410-1-ap	IHC: 1:1000<br>IF: 1:2500	Proteintech, CHINA
ACTN3	GB113424	IHC: 1:1500<br>IF: 7500	Servicebio, CHINA
NEFH	18934-1-AP	IF: 1:1000	Proteintech, CHINA
DGKZ	GB113428	IF: 1:3000	Servicebio, CHINA

Supplementary Table 2. PCR-primers

Gene	Sequence（5'--3'）		
R-ACTIN-F	CGTTGACATCCGTAAAGACCTC		
R-ACTIN-R	TAGGAGCCAGGGCAGTAATCT		
R-Rab3a-F1	TACATATGGCCTCAGCCACAG	SJA	AS
R-Rab3a-R1	GTTTTGCCCACACTGCTGTT	SJA	AS
R-Rab3a-F2	CCTCACCGATGGCCAGA	SJB	MODEL
R-Rab3a-R2	TCGTCTGCGTAGCGGAAAAG	SJB	MODEL
R-Ank3-F1	TGGTCCACAGAGTCCTTGTGA	SJA	AS
R-Ank3-F2	TCCCTAGCCTTACGTCCACA	SJB	MODEL
R-Ank3-R	CGGCTACTATTGCCATCCTG	SJB	
R-Shank2-F1	AGGCAGAAATCGATAGGGATAAC	SJA	AS
R-Shank2-R	CATGGACAAGCTTCGGGTGA		
R-Shank2-F2	CTCGAGGGATAACAGAGGAAGA	SJB	MODEL
R-Filip1-R	GAGGACTTGGGGCAGGAGAC		
R-Filip1-F1	AAGCCTGCTGTTTGCTGTCTG	SJA	AS
R-Filip1-F2	CAAGGGATAAACCAGCGGC	SJB	MODEL
R-Grid2-F	GAGGAGCCCTGGACATCAAG		
R-Grid2-R1	TTTATGTGACAAATCTTTCAGGAC	SJA	AS
R-Grid2-R2	AGTCCCATCGAAAGAGGCTC	SJB	MODEL
R-Lrrc4c-F	TTTTGTGCCTCAGAGAATCC		
R-Lrrc4c-R2	CAACATGAACAAACCTGTCTTC	SJA	AS
R-Lrrc4c-R1	GTGTTTGCCAACCTGTCTTC	SJB	MODEL
R-Igsf9b-F1	CTATCTGGGCAGTGTTGTCG	SJA	AS
R-Igsf9b-R	GGTATCTCAGAGCCTCCAGC		
R-Igsf9b-F2	CCTTACCTACTTCAGGAATACTTC	SJB	MODEL

R-Dgkz-F1	ACCCTGCTTATGTAGGAAAGCC	SJA	AS
R-Dgkz-R1	CACAGTACTCCGGATCTGCC	SJA	AS
R-Dgkz-F2	AGGTTCCCCAGACACAGAAAG	SJB	MODEL
R-Dgkz-R2	CGCTCAGATTCACCGCACG	SJB	MODEL
