# Supplementary material for: Comprehensive profiling and molecular characterization of alternative splicing regulation in synaptic remodelling associated with neuropathic pain induced by chronic constrictive injury in a rat model
Source: RNA Biol. 2026 May 15;23(1):1–18. doi: 10.1080/15476286.2026.2675080 (PMC13240960; doi:10.1080/15476286.2026.2675080)
Supplement: Supplemental Material [file KRNB_A_2675080_SM3593.pdf]

Figure S1. Identification and characterization of alternative splicing events in spinal cord samples from a chronic constrictive injury rat model.

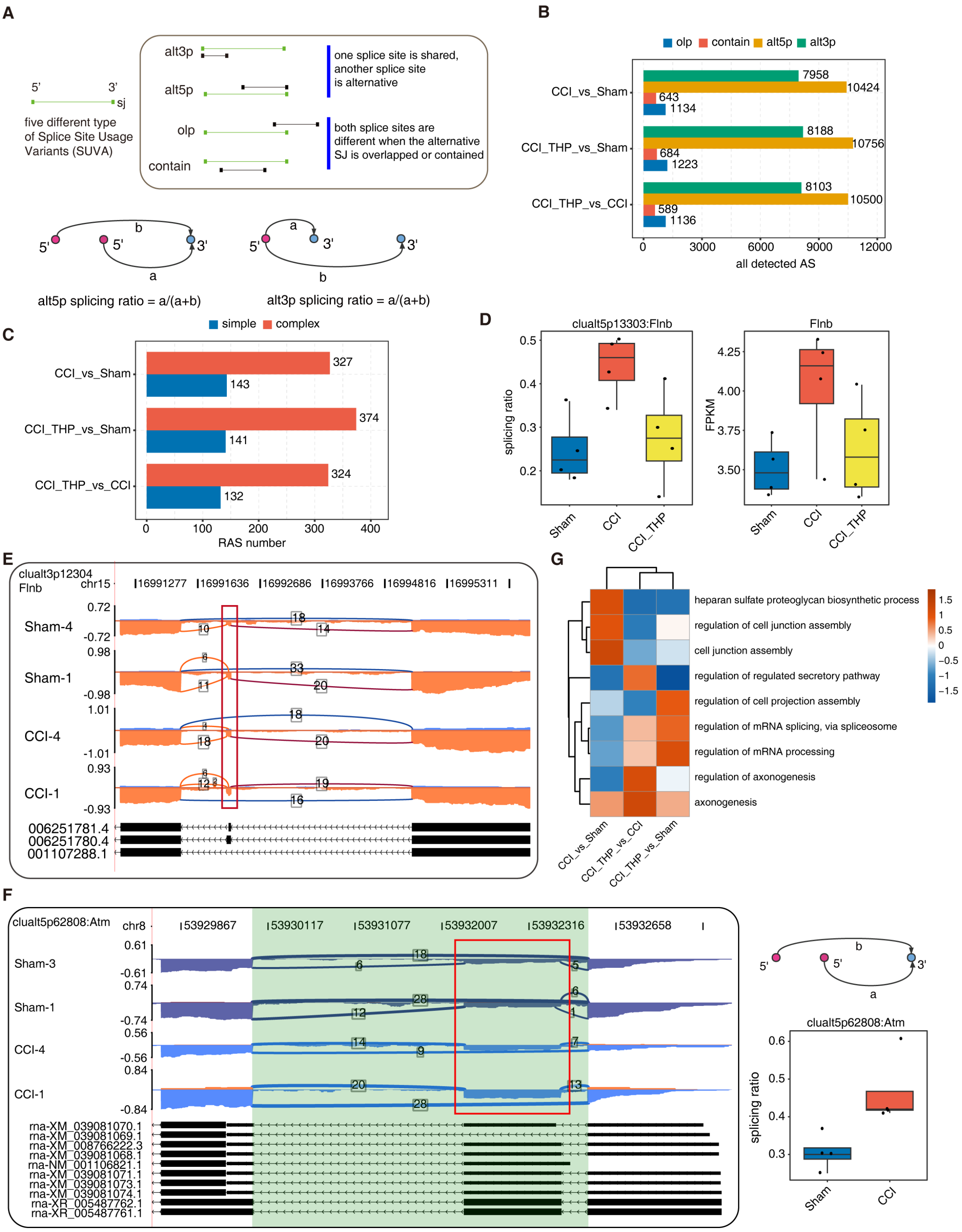

A. Four different types of AS event model defined by SUVA according to splicing site usage variation. Each type contains two paired splice junctions (SJ). “alt3p” indicates model that 5’ splice site is shared and 3’ splice site is alternative. “alt5p” indicates model that 3’ splice site is shared and 5’ splice site is alternative. “olp” indicates model that both splice sites are different but part of the splice junction are overlapped. “contain” indicates model that both splice sites are different but one splice junction is

contained in another splice junction. Alt3p and alt5p events were resolved into a directed acyclic graph.

B. Bar plot showing all detected AS events by SUVA among Sham, CCI, and CCI_THP samples.

C. Bar plot showing the number of SUVA RAS events that contains SJs involved in two or more different classical splicing events (complex) or in the same classical splicing event (simple).

D. Boxplot showing splicing ratio of RAS clualt5p13303:Flnb and expression profile of its hostgene in Sham, CCI, and CCI_THP samples.

E. Visualization of reads distribution of Flnb in AS events clualt5p13303 from different groups. Splice junctions were labelled with SJ reads number. And altered exon was marked out with box. Boxplot in the right panel showing splicing ratio profile of Sham and CCI samples of the splicing event from Flnb showed in left panel.

F. Visualization of reads distribution of Atm in AS events clualt5p62808 from different groups. Splice junctions were labelled with SJ reads number. And altered exon was marked out with box. Boxplot in the right panel showing splicing ratio profile of Sham and CCI samples of the splicing event from Atm showed in left panel.

G. Gene ontology enrichment analysis of biological processes of RASGs in each comparing groups. Top 3 terms were selected for each cluster and heatmap shows the enrichment p-value of these terms (scaled by column).

Figure S2. Alternative splicing of genes associated with synaptic formation and remodeling undergoes significant regulation during the onset of neuropathic pain.

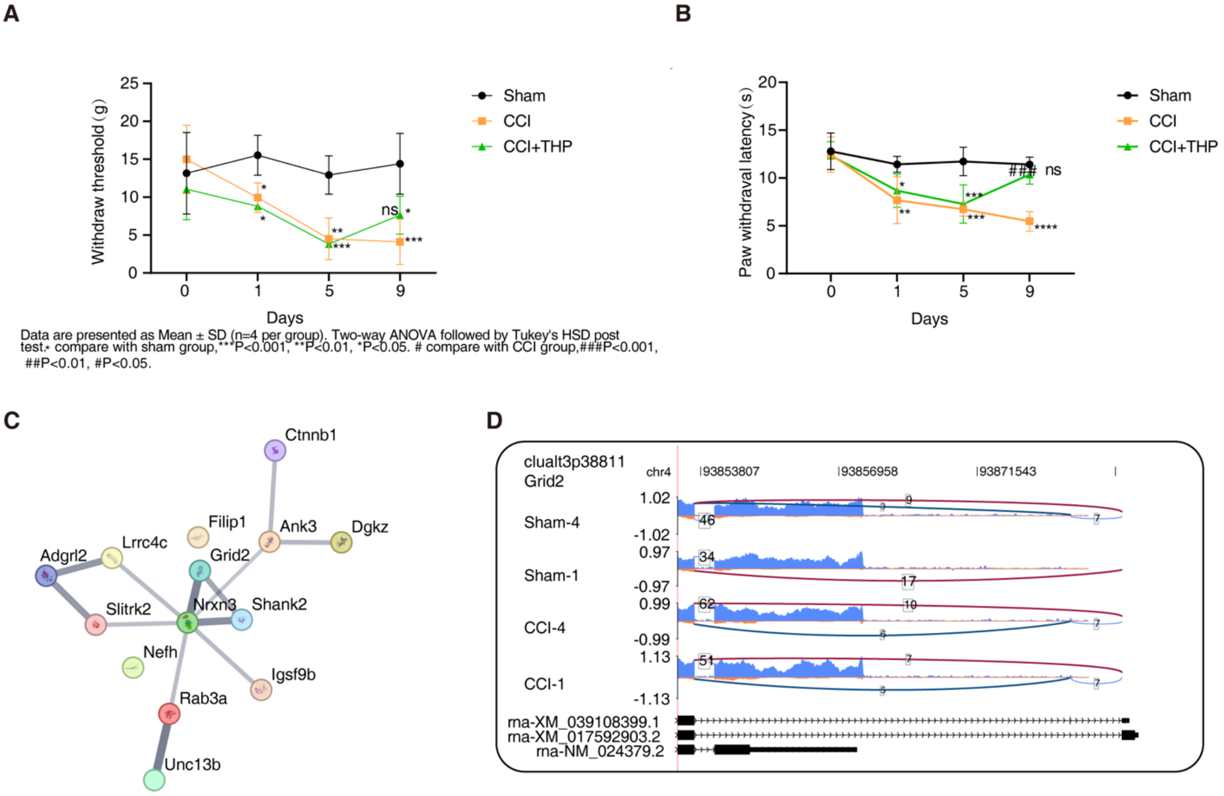

A. Mechanical withdrawal threshold (MWT) was assessed using Von Frey filaments at baseline (day 0) and on days 1, 5, and 9 post-surgery. CCI surgery significantly induced mechanical allodynia, while THP treatment effectively increased the withdrawal threshold.

B. Thermal paw withdrawal latency (PWL) was measured using the Hargreaves test. THP treatment significantly reversed CCI-induced thermal hyperalgesia, with latencies returning to levels comparable to the Sham group by day 9.

C. PPI network diagram showing protein interaction of genes involving synaptic plasticity related RAS comparing CCI and Sham samples.

D. Visualization of reads distribution of Grid2 in AS events clualt3p38811 from different groups. Splice junctions were labelled with SJ reads number. And altered exon was marked out with box. Boxplot in the right panel showing splicing ratio profile of Sham and CCI samples of the splicing event from Grid2 showed in left panel.

Figure S3. Levo-tetrahydropalmatine treatment restores aberrant splicing patterns of synaptic remodeling-related genes.

A

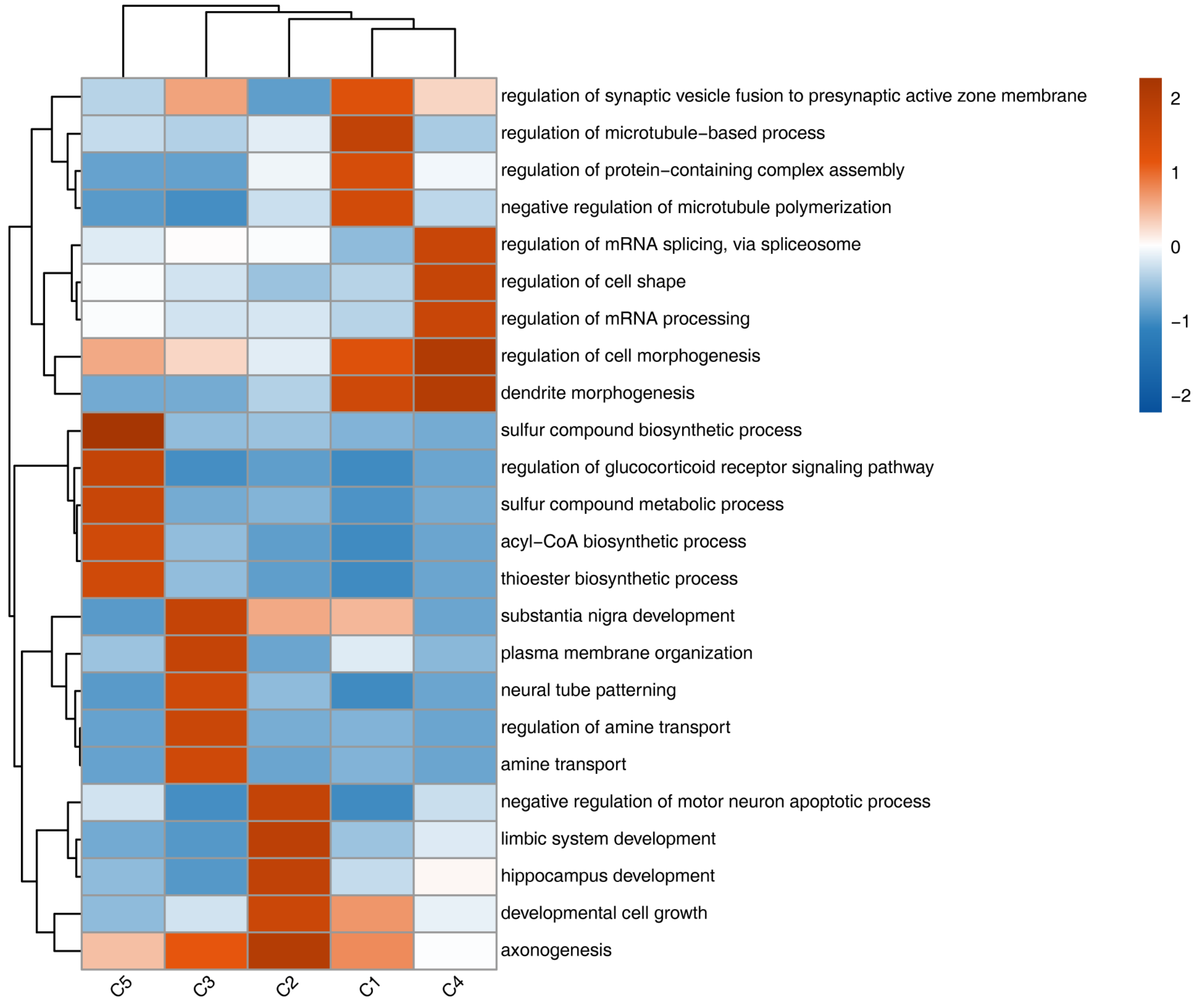

A. Gene ontology enrichment analysis of biological processes of RASG in each cluster using kmeans. Top 5 terms were selected for each cluster and heatmap shows the enrichment p-value of these terms (scaled by column).

Figure S4. Construction of a co-regulatory network for RBPs and RAS events reveals potential mechanisms in neuropathic pain.

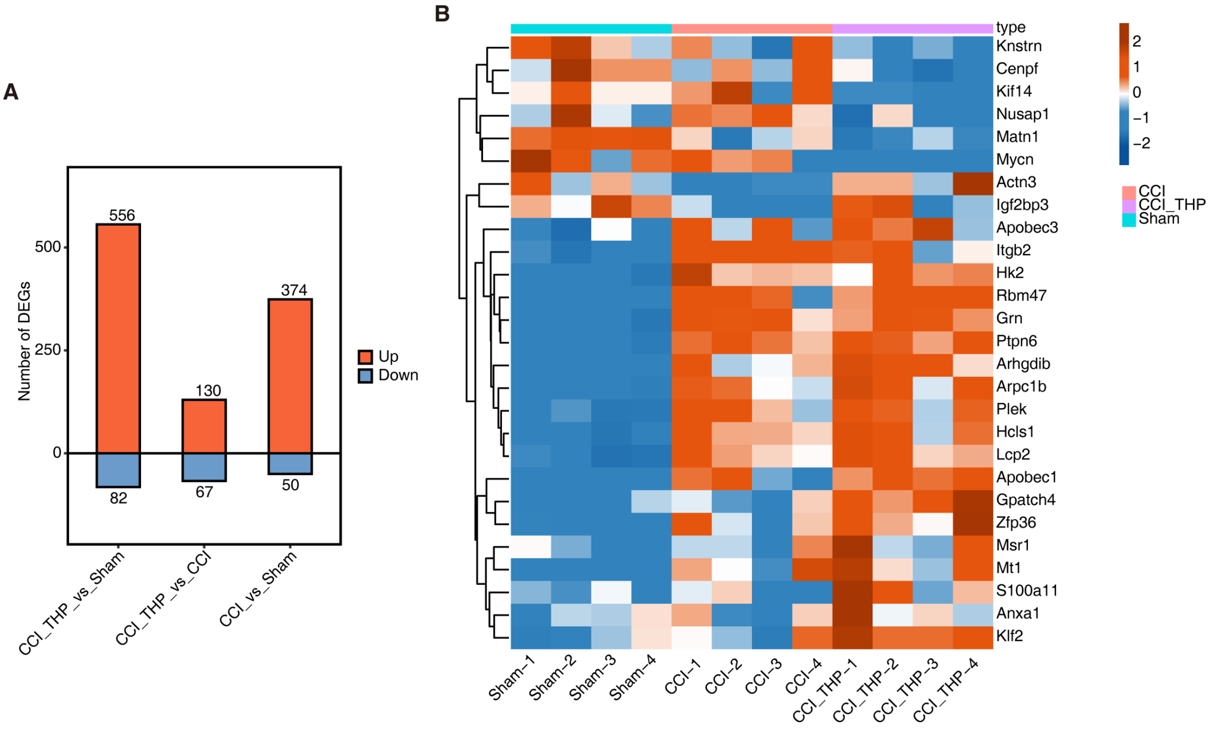

A. The number of differentially expressed (DE) genes among different groups. Bar plot showing the number of up-regulated and down-regulated DEGs.

B. The Heatmap diagram showing the expression profile of DE RBPs.
